# A novel 2,5-diaminopyrimidine-based affinity probe for Bruton’s tyrosine kinase

**DOI:** 10.1038/srep16136

**Published:** 2015-11-04

**Authors:** Yingying Zuo, Yanxia Shi, Xitao Li, Yingqi Teng, Zhengying Pan

**Affiliations:** 1 Key Laboratory of Chemical Genomics, Key Laboratory of Structural Biology, School of Chemical Biology and Biotechnology, Shenzhen Graduate School, Peking University, Shenzhen, China 518055; 2 Shenzhen Reciproca Pharmaceuticals Co., Ltd., Xili University Town, Shenzhen, China 518055

## Abstract

As a critical regulator of the B-cell receptor signaling pathway, Bruton’s tyrosine kinase (Btk) has attracted intensive drug discovery efforts for treating B-cell lineage cancers and autoimmune disorders. In particular, covalent inhibitors targeting Cys481 in Btk have demonstrated impressive clinical benefits, and their companion affinity probes have been crucial in the drug development process. Recently, we have discovered a novel series of 2,5-diaminopyrimidine-based covalent irreversible inhibitors of Btk. Here, we present the discovery of a novel affinity Btk probe based on the aforementioned scaffold and demonstrate its usage in evaluating the target engagement of Btk inhibitors in live cells.

Bruton’s tyrosine kinase (Btk) is a cytosolic non-tyrosine kinase that is expressed only in hematopoietic cells, except in natural killer and T cells. Btk participates in several signaling pathways, particularly in the B cell receptor (BCR) pathway, which is crucial in B-cell development and differentiation^1^. In cells, Btk is activated by its upstream kinases through the phosphorylation of a tyrosine residue (Tyr551), followed by the autophosphorylation of another tyrosine residue (Tyr223). The fully activated Btk then phosphorylates its substrates, including PLC-γ 2 in the BCR pathway. Extensive *in vivo* and clinical studies strongly suggest that Btk is involved in the development of multiple B-cell malignancies and autoimmune diseases such as rheumatoid arthritis and lupus^2^. Multiple Btk inhibitors have been developed (Fig. 1a). Ibrutinib^3^ (CRA-032765, PCI-32765, Imbruvica®), a covalent irreversible inhibitor from Celera/Pharmacyclics/Janssen, became the first clinically approved Btk-targeting drug in November 2013. CC-292 (AVL-292)^4^ from Celgene is the second covalent irreversible inhibitor that is currently undergoing clinical trials. Both ibrutinib and CC-292 form a covalent bond with a cysteine residue (Cys481) located at the rim of the ATP-binding pocket in Btk. Other clinical-stage Btk inhibitors include a compound from ONO Pharmaceutical and PRN1008/HM71224 from Hanmi Pharmaceutical^5^,^6^. GDC-0834, a non-covalent reversible Btk inhibitor from Gilead/Roche, was evaluated in a Phase I clinical trial, but no recent developments have been reported^7^.

Target engagement refers to the occupancy of intended biological targets by drug molecules^8^. This information is crucial for building a correlation between phenotypic observations and inhibitor-biomolecule interactions at the molecular level. Targeted covalent drugs^9^,^10^, due to their inherent reactive groups, are particularly suitable for developing small molecule affinity probes that may be used to measure the extent of target occupancy. PCI-33380 was designed based on the ibrutinib scaffold and has been used in both cellular and *in vivo* studies that demonstrated the connection between the inhibitor binding event and phenotypic readouts of cellular responses due to the inhibition of Btk functions^11^. Furthermore, the use of fluorescent probes in clinical trials has played an important role in determining the appropriate dosage of drugs for patients^12^. In addition to PCI-33380, other fluorescent probes for Btk that also utilize the ibrutinib scaffold have been recently reported for the imaging of Btk in live cells^13^,^14^ (Fig. 1b).

As depicted in Fig. 2a, affinity probes normally include three components: a recognition group, a reactive group and a reporting group. The recognition group directs the probe into the binding pocket of the targeted protein and facilitates the formation of a covalent bond between the reactive group and the biomolecule. The reporting group provides a convenient means of identifying probe-bound proteins within complex proteomes. Figure 2b shows a general scheme of assays to examine the target engagement of drug molecules. By sequentially adding inhibitors and probes into biological samples (cells, tissues, etc.), the intensities of probe-labelled bands will give a direct readout of those biological targets are not occupied by inhibitors. As the concentration of inhibitors increases, a decrease of band intensity indicates a portion of biological targets are engaged by inhibitors.

Recently, we discovered a novel series of Btk covalent inhibitors based on the 2,5-diaminopyrimidine scaffold^15^. Herein, we present our efforts in developing that series of inhibitors into a novel affinity Btk probe. The resulting probe selectively labeled Btk and provided an efficient method of directly measuring the target engagement of Btk inhibitors in live cells.

## Chemistry

**Figure 3:**
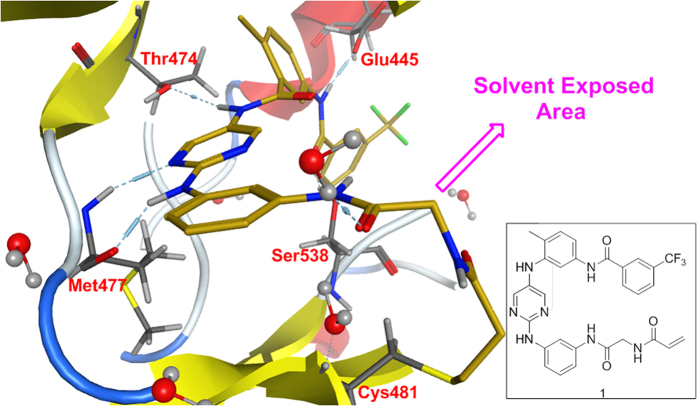
Modeling of compound **1** (carbon atoms in yellow) in Btk’s active site (PDB code 3PJ3). The light blue dots indicate potential hydrogen bonds between compound **1** and Btk. Cys481 is at the C-terminus of the hinge region.

A 2,5-diaminopyrimidine compound (**1**) was smoothly docked into a crystal structure of Btk (PDB ID: 3PJ3) without obvious steric conflicts by visual inspection (Fig. 3). While covalently linked to the sulfhydryl group of Cys481, compound **1** exhibited an extended conformation, forming important hydrogen bonds with several residues in Btk, from Met477 at the hinge region and the gatekeeper residue Thr474 to Glu445 and Ser538 at the DFG-out pocket. In particular, the glycyl moiety was selected for substitution by other groups as a suitable point of attachment for the reporting group.

**Figure 4:**
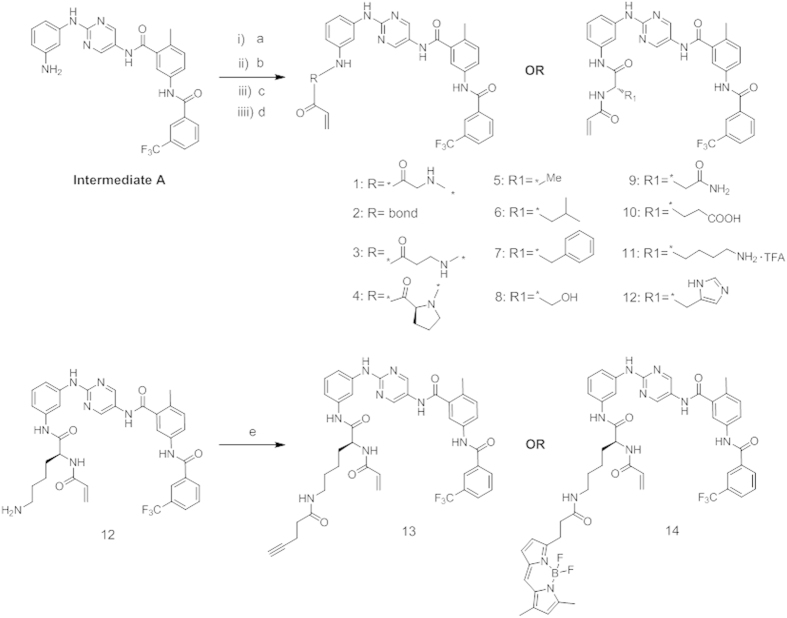
Preparation of compounds **1–14**. Reagents and conditions: (a) O-(7-azabenzotriazol-1-yl)-N,N,N’,N’-tetramethyluroniumhexafluorophosphate (HATU), diethylpropylamine (DIEA), amino- and side chain functional group-protected amino acids, DMF, 0 °C to rt, overnight (80–96%) or EDCI, HOBt, Et_3_N, DMF, 0 °C to rt, overnight (80%) ((a) acryloyl chloride, THF, H_2_O, 0 °C to rt, 2–5 h (80-90%) for compound **1**). (**b**) TFA, DCM, rt, 2 h (90–98%) or morpholine, DMF, rt, overnight (80–95%). (c) Acryloyl chloride, THF, H_2_O, 0 °C to rt, 2–5 h (80–90%). (d) TFA, DCM, rt, 2 h (90–98%); TFA, several drops of water, rt, 3 h (80%) for compound 8; or HOBT, THF, rt, overnight (75%) for compound 12. (e) HATU, DIEA, pent-4-ynoic acid or BODIPY™ FL, DMF, 0 °C to rt, overnight (70–85%).

The synthesis of compounds is depicted in Fig. 4. Intermediate **A**, which has been previously reported in the literature, was used as the starting compound^15^. Common amide bond formation and protection group manipulation yielded compounds **1–14**. The coupling of the lysine derivative **11** with pent-4-ynoic acid or BODIPY™ FL acid generated **13** and **14**, respectively. The reactions proceeded smoothly, with 70–98% yields.

## Results and Discussion

### Structure-activity relationship

We focused our efforts on probing the space around the glycyl moiety in **1** by replacing it with several amino acid residues [Table 1]. Small side chains were generally preferred because β-amino propionic acid (**3**), alanyl (**5**), and serinyl (**8**) substitutions all generated potent inhibitors. Increasing the size of the side chain groups at R1 gradually increased the IC_50_ values from 50–60 nM (**4**, **11**, **12**) to more than 100 nM (**6** and **7**). Interestingly, similar to compound **8**, compounds **9** and **10** with polar group substitutions maintained their potency (15–20 nM), suggesting possible H-bonding interactions with the protein.

The Lys derivative **11** demonstrated acceptable inhibition, with an IC_50_ of 63 nM. Compound **13**, in which the terminal amino group was capped with a pent-4-ynoic acid, exhibited an apparent IC_50_ of 38 nM. We further measured the k_inact_/Ki values of three inhibitors. Compound **2** is a potent inhibitor with k_inact_/Ki value of 1.83 ± 0.41 × 10^5^ M^−1^s^−1^, while those values of compound **11** and **13** are 1.35 ± 0.04 × 10^5^ M^−1^s^−1^ and 0.75 ± 0.08 × 10^5^ M^−1^s^−1^, respectively. These data indicated that extending a reporting group from the side chain of lysine residue largely maintained potency, thus the lysine moiety was chosen as the anchor point for the attachment of a fluorescent group to generate probe **14**.

### Compound 14 is a selective affinity probe for Btk

**Figure 5:**
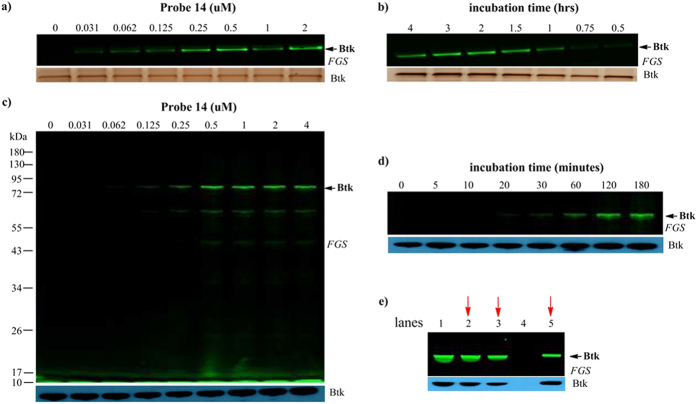
Probe **14** is a selective affinity probe for Btk. (**a**) Concentration-dependent labeling of recombinant Btk by probe **14**; (**b**) time-dependent labeling of recombinant Btk by probe **14**; (**c**) probe **14** predominantly labeled endogenous Btk in live cells (concentration course); (**d**) time course experiments in cellular labeling; (**e**) immunoprecipitation of Btk from probe **14**-labeled lysates. lane 1: cell lysates; lane 2: supernatant after removal of intrinsic IgG; lane 3: supernatant after immunoprecipitation; lane 4: supernatant of the last wash before elution; lane 5: supernatant of the first elution by applying LDS sample buffer onto protein A Sepharose beads.

We first tested whether probe **14** could label Btk. When recombinant Btk (0.5 μg) was incubated with increasing concentrations of probe **14**, fluorescent signals increased accordingly, and 0.5 μM was selected as the probe concentration for the next steps because it already provided a sufficiently strong signal. When probe **14** was incubated with Btk for increasing time periods, the brightness of the fluorescent signals reached a maximum at 2 hours (Fig. 5a,b).

Next, we examined the labeling properties of probe **14** in live cells. Because Btk is expressed in all hematopoietic cells except T and natural killer cells, OCI-Ly7 cell, a B-cell line, was used in the study. A dominant band was present at the expected molecular weight of Btk (approximately 76 kDa), with a couple of minor bands at lower molecular weights. This dominant band was also immunoreactive against anti-Btk antibody. Concentration course experiments indicated again that the Btk band intensity reached saturation at 0.5 μM probe **14**. Time course experiments also suggested that an incubation time of 2 hours was sufficient for labeling (Fig. 5c,d). To further confirm that Btk was indeed labeled by probe **14** in these cells, Btk was successfully immunoprecipitated from probe **14-**labeled lysates (Fig. 5e). Taken together, Btk was indeed the dominant band labeled by probe **14** in OCI-Ly7 cells. As expected, no significant labeling was detected in Jurkat cells, a T-cell line that does not express Btk (Supplementary Figure S1). Comparing to PCI-33380 (Supplementary Figure S2), probe **14** has a slower saturation rate that may be due to its structure resembling to Type-II kinase inhibitors.

### 2,5-Diaminopyrimidine series of inhibitors directly engage Btk in live cells

**Figure 6:**
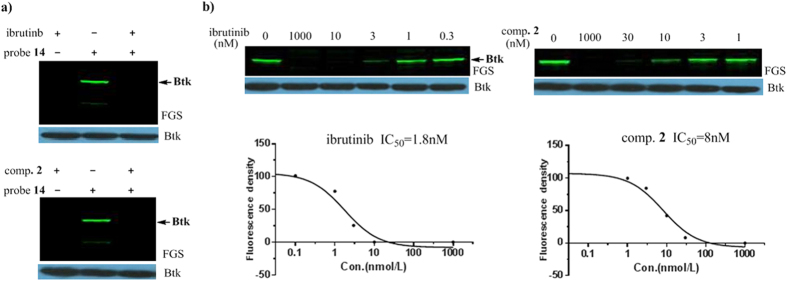
Target engagement of Btk inhibitors: (**a**) labeling of Btk by probe **14** (0.5 μM) was completely competed off by ibrutinib and compound **2** (1 μM); (**b**) measurement of the extent of Btk occupancy by inhibitors (ibrutinib and compound **2**) in live cells. Band densitometry was measured by Gelpro32, and Graphpad Prism was used to determine the IC_50_ values.

After the labeling conditions were optimized, we examined whether probe **14** could be used to access the target engagement of inhibitors towards Btk. Two types of structurally different Btk inhibitors were examined: the clinically approved Btk drug ibrutinib and compound **2**, which contains the same scaffold as probe **14**. Cells were first incubated with the inhibitors for 1 hour, followed by 2 hours incubation with 0.5 μM of probe **14**. As shown in Fig. 6a, both compounds at 1 μM effectively blocked the labeling of Btk by probe **14**. To measure the extent of Btk occupancy by the inhibitors in live cells, OCI-Ly7 cells were incubated with increasing concentrations of the compounds for 1 hour prior to labeling with probe **14** for 2 hours. After cell lysis, the protein contents were directly loaded onto gels. After electrophoresis, the bands’ fluorescent densities were measured. As presented in Fig. 6b, the IC_50_ values for Btk occupancy by ibrutinib and compound **2** were 2 nM and 8 nM, respectively, which are generally in-line with those measured with PCI-33380 (3 nM and 21 nM, Supplementary Figure S2). These cellular occupancy data are in good accordance with their IC_50_s in enzymatic assays, and clearly demonstrate that covalent inhibitors can maintain high potency in live cells, unlike reversible kinase inhibitors, which face competition by intrinsic cellular ATP and often exhibit a reduction in their inhibitory potency against kinases in cellular assays compared with biochemical assays.

## Conclusions

The development of companion affinity probes is a crucial component of drug discovery programs of targeted covalent kinase inhibitors. Recently, we disclosed a novel 2,5-diaminopyrimidine-based series of Btk inhibitors that demonstrate excellent potency in both cellular and *in vivo* experiments. In this study, we present the development of a novel fluorescent Btk probe. The SAR study resulted in the identification of an anchor point to which a bulky fluorescent group could be linked. After appropriate labeling conditions were identified, this probe was successfully applied to measure the target occupancy rate of Btk inhibitors in native cells. The approach described here could be applied in future studies that require the establishment of a correlation between the pharmacological effects of small molecule inhibitors and their target occupancy rates.

## Methods

### Chemistry

All of the reagents were purchased commercially and used without further purification, unless otherwise stated. All yields refer to the chromatographic yields. Anhydrous dimethyl formamide (DMF) was distilled from calcium hydride. Brine refers to a saturated solution of sodium chloride in distilled water. Reactions were monitored by thin-layer chromatography (TLC) carried out on 0.25 mm Yantai silica gel plates (HSGF254) using UV light as the visualizing agent. Flash column chromatography was carried out using Yantai silica gel (ZCX-II, particle size 0.048–0.075 mm). ^1^H-NMR and ^13^C-NMR spectra were recorded on a Bruker Advance 400 (^1^H: 400 MHz, ^13^C: 100 MHz) or Bruker Advance 300 (^1^H: 300 MHz, ^13^C: 75 MHz) spectrometer at ambient temperature, with the chemical shift values presented as ppm relative to TMS (*δ*_H_ 0.00 and *δ*_C_ 0.00), dimethyl sulfoxide (*δ*_H_ 2.50 and *δ*_C_ 39.52), or methanol (*δ*_H_ 3.31 and *δ*_C_ 49.00) standards. The data are reported as follows: chemical shift, multiplicity (s = singlet, d = doublet, t = triplet, q = quartet, br = broad, m = multiplet), coupling constants and number of protons. HR-MS spectra were obtained using a Bruker Apex IV RTMS instrument. Compound purity was determined based on HPLC chromatograms acquired on an Agilent 1200 HPLC or 1260 HPLC. The analyses were conducted on an Agilent PN959990-902 Eclipse Plus C18 250 mm × 4.6 mm column, using a water-MeCN gradient with MeCN from 50% to 98% or 50% to 65% over 10 min. Detection was performed at 254 nm, and the average peak area was used to determine purity. All of the compounds were determined to be >95% pure. **(Detailed synthetic routes are available in the supporting information).**

### Modeling study

MOE 2013 was used to perform the modeling study. We first drew compound **1** using the Builder function of MOE, followed by the generation of a molecular conformation library, which was later used for docking. The protein template (PDB:3PJ3) was loaded into MOE, and the structure was prepared and docked with the molecular conformation library. The optimal structure was obtained using the scoring function of MOE. Then, a C-S bond between the S atom of Cys_481_ and the terminus C_sp2_ of compound **1** was manually added while altering the C_sp2_ carbon to C_sp3_, further geometry optimization yielded the covalent docking structure.

### Biological assays

#### Kinase enzymology assays

Kinases were purchased from Carna Biosciences. Kinase enzymology assays were performed according to the protocols specified for the HTRF® KinEase™ assays sold by Cisbio Bioassays.

#### Kinetic study of Btk inhibitors

The kinase assays were performed at room temperature. The compounds with serial dilution in DMSO were added into reaction buffer with 0.5 nM Btk, incubating with different periods of time (0 min, 4 min, 8 min, 12 min, 16 min, and 20 min). Enzyme reaction was started by adding ATP and substrate to the reaction mixture. The enzyme activity was measured with HTRF® KinEase™ assays. The data analysis was guided by the book, Enzyme Kinetics by Hans Bisswanger^16^.

#### Recombinant protein labeling assays

In 25 μL of PBS buffer, 0.5 μg of recombinant Btk was incubated with increasing concentrations of probe **14** for 2 h and then analyzed by SDS/PAGE and fluorescent gel scanning (fluorescence, CY2). The gel was then blotted, and the total Btk levels were detected by standard silver staining. The concentration course labeling procedure was similar to that for the time course labeling.

#### Cellular labeling assays

Labeling of Btk by probe **14**. A total of 1.5 × 10^6^ cells were treated with probe **14** at 1 μM for different lengths of times (5 min, 10 min, 20 min, 30 min, 1 h, 2 h, 3 h, or 4 h), washed, lysed in cell lysis buffer (Beyotime) containing 1 mM PMSF and 10 mM NaF (Invitrogen), and centrifuged. The sample protein concentrations were quantified using a NanoDrop 2000 spectrophotometer, and the samples were adjusted to the same concentration then analyzed by SDS/PAGE and fluorescent gel scanning (fluorescence, CY2). The gel was then blotted, and the total Btk levels were detected by a standard Western blot. The concentration course labeling procedure was similar to that for the time course labeling.

Immunoprecipitation of Btk. LY7 cells were treated with probe **14** at 0.5 μM for 2 h, lysed in binding buffer (20 mM Na_3_PO_4_, pH 7.5, 150 mM NaCl) containing phosphatase inhibitors and protease inhibitors. Obtained lysates was preincubated with Protein A Sepharose beads (GE healthcare, 17-5138-01) to remove intrinsic cellular IgG proteins. Meanwhile, anti-Btk (CST, 8574S) from rabbit was preincubated with Protein A Sepharose beads for 2 hours at 4 °C. Then, the pre-treated lysates were added to the pre-treated immobilized Protein A Sepharose, incubated for 2 hours at 4 °C. The immune complexes were washed with binding buffer for four times and eluted with LDS sample buffer (50 mM Tris-HCl, 2% SDS, 0.1% bromophenol blue, 10% glycerol, 1% DTT) and analyzed by Western blot, as described above.

Competition assay. LY7 cells were preincubated with the compounds (1 μM) for 1 h before labeling with probe **14** under the proper time and concentration conditions. Then, the cells were lysed and analyzed as described above.

Target engagement of Btk inhibitors. LY7 cells were preincubated with different concentrations of compounds for 1 h before labeling with probe **14**. Then, the samples were lysed and analyzed as described above. Gelpro32 software was used to analyze the Btk band density to obtain the half-maximum active site occupancy values.

## Additional Information

**How to cite this article**: Zuo, Y. *et al.* A novel 2,5-diaminopyrimidine-based affinity probe for Bruton’s tyrosine kinase. *Sci. Rep.* **5**, 16136; doi: 10.1038/srep16136 (2015).

## Supplementary Material

Supplementary Information

## Figures and Tables

**Figure 1:**
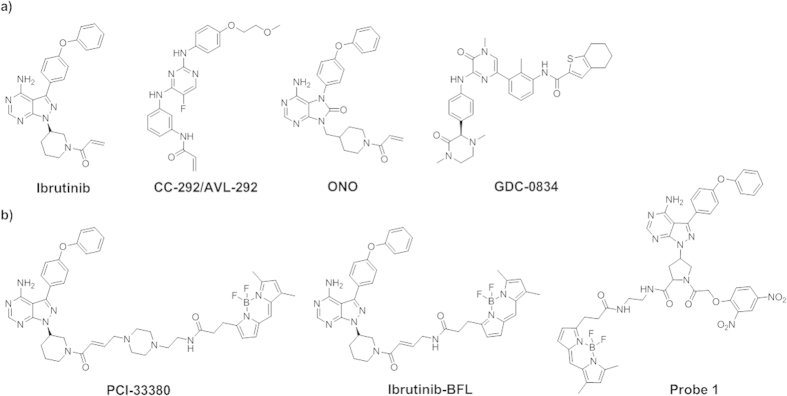
Structures of representative Btk (**a**) inhibitors and (**b**) fluorescent probes.

**Figure 2:**
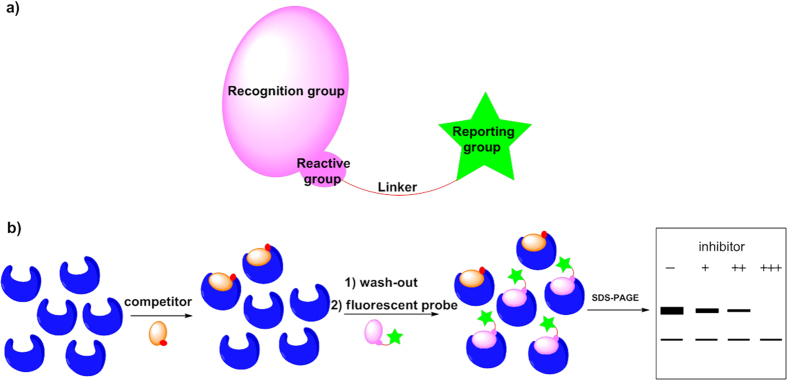
(**a**) Components of affinity probes; (**b**) general scheme of measuring target engagement by competition assays between inhibitors and affinity probes.

**Table 1:**
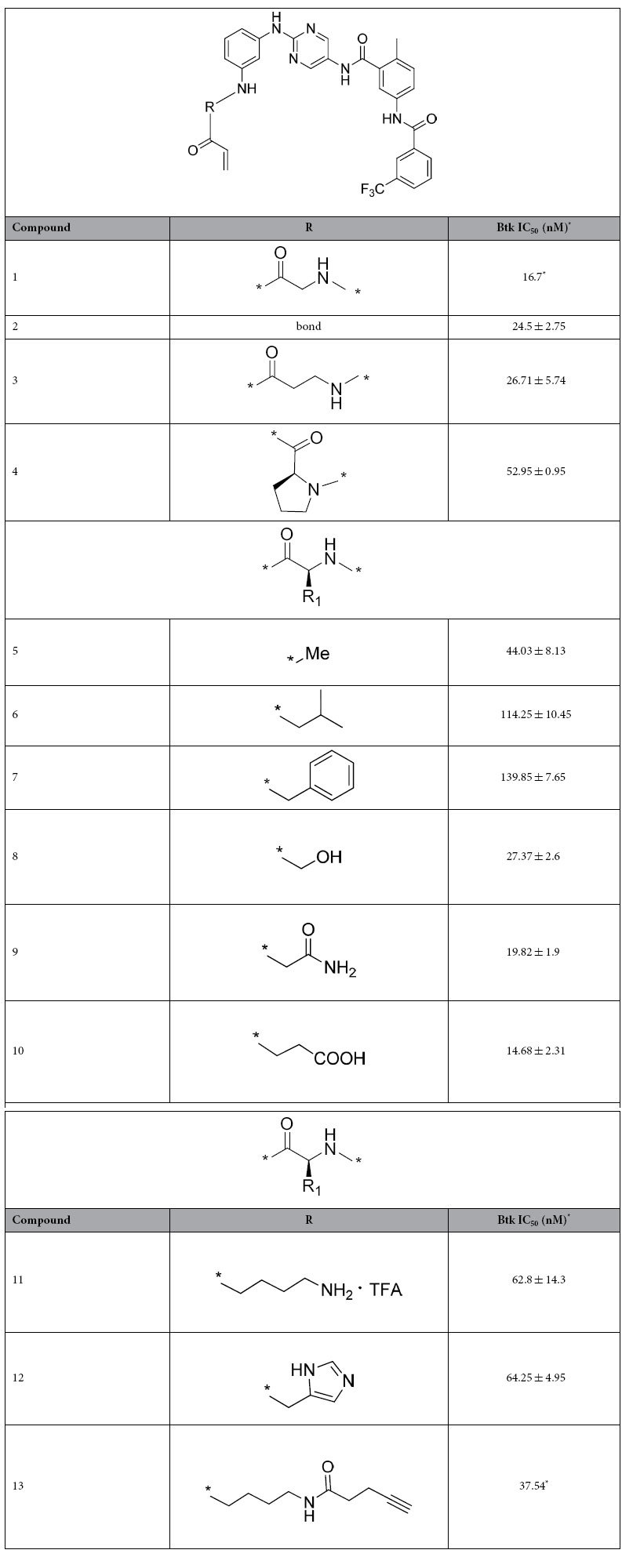
Structure-activity relationship of Btk inhibitors.

^*^The values are determined by a single run of duplicates and all others are means of two individual measurements.
