# Supplementary material for: A novel 2,5-diaminopyrimidine-based affinity probe for Bruton’s tyrosine kinase
Source: Sci Rep. 2015 Nov 4;5:16136. doi: 10.1038/srep16136 (PMC4648318; doi:10.1038/srep16136)
Supplement: Supplementary Information [file srep16136-s1.doc]

**Supporting Information**

**A novel 2,5-diaminopyrimidine-based affinity probe for Bruton’s tyrosine kinase**

**Yingying Zuo^a^, Yanxia Shi ^b^, Xitao Li ^a^, Yingqi Teng ^b^, Zhengying Pan^a^,***

^a^Key Laboratory of Chemical Genomics, Key Laboratory of Structural Biology, School of Chemical Biology and Biotechnology, Shenzhen Graduate School, Peking University, Shenzhen, China 518055

^b^Shenzhen Reciproca Pharmaceuticals, Co. Ltd. Xili University Town, Shenzhen, China 518055

*Corresponding author. Tel.:+8675526033072

*E-mail address*: panzy@pkusz.edu.cn

**Table of Contents**

1. General Information page 2

2. Synthetic Routes of compounds 1-14 pages 3

3. Experimental Procedures and Spectroscopic Data pages 4-9

4. NMR and HPLC Spectra pages 10-26

5. Biological data pages 26-27

1. **General Information**

All the reagents were purchased commercially and used without further purification, unless otherwise stated. All yields refer to chromatographic yields. Anhydrous dimethyl formamide (DMF) was distilled from calcium hydride. Brine refers to a saturated solution of sodium chloride in distilled water. Reactions were monitored by thin-layer chromatography (TLC) carried out on 0.25 mm Yantai silica gel plates (HSGF254) using UV light as visualizing agent . Flash column chromatography was carried out using Yantai silica gel (ZCX-II, particle size 0.048-0.075 mm). ^1^H-NMR and ^13^C-NMR spectra were recorded on a Bruker Advance 400 (^1^H: 400 MHz, ^13^C: 100 MHz ) or Bruker Advance 300 (^1^H: 300 MHz, ^13^C: 75 MHz) spectrometer at ambient temperature with chemical shift values in ppm relative to TMS (*δ*_H_ 0.00 and *δ*_C_ 0.00), dimethyl sulfoxide (*δ*_H_ 2.50 and *δ*_C_ 39.52), or methanol (*δ*_H_ 3.31 and *δ*_C_ 49.00) as standard. Data are reported as follows: chemical shift, multiplicity (s = singlet, d = doublet, t = triplet, q = quartet, br = broad, m = multiplet), coupling constants and number of protons. HR-MS were obtained using Bruker Apex IV RTMS. Purity of compounds was determined by HPLC chromatograms acquired on an Agilent 1200 HPLC or 1260 HPLC. Analyses were conducted by an Agilent PN959990-902 Eclipse Plus C18 250 mm × 4.6 mm column, using a water–MeCN gradient with MeCN from 50% to 98% or 50% to 65% in 10 min. Detection was at 254 nm, and the average peak area was used to determine purity. All the compounds were determined to be >95% pure.

1. **Synthetic Routes of compounds 1-14**

The synthesis of new affinity probes were depicted below. It started from a literature known compound **intermediate A**. Common amide bond formation and protection group manipulation gave compounds **1-14**. Coupling lysine derivative **11** with pent-4-ynoic acid or BODIPY™ FL acid provided two types of probes **13** and **14** respectively. Reactions generally proceeded smoothly with 70-98% yields.

^a^Reagents and conditions: (a) HATU, DIEA, amino- and side chain functional group- protected amino acid, DMF, 0^o^C~rt, overnight(80-96%) or EDCI, HOBt, Et_3_N, DMF, 0^o^C~rt, overnight(80%) ((a) acryloyl chloride, THF, H2O, 0^o^C~rt, 2-5h(80-90%) for compound1). (b) TFA, DCM, rt, 2h(90-98%) or morpholine, DMF, rt, overnight(80-95%). (c) acryloyl chloride, THF, H2O, 0^o^C~rt, 2-5h(80-90%). (d) TFA, DCM, rt, 2h(90-98%); or TFA, several drops of water, rt, 3h(80%) for compound 8; or HOBT, THF, rt, overnight(75%) for compound 12. (e) HATU, DIEA, pent-4-ynoic acid or BODIPY™ FL, DMF, 0^o^C~rt, overnight(70%-85%).

1. **Experimental Procedures and Spectroscopic Data**

**Preparation of compound 3-7**

Synthesis of (S)-N-(2-((3-(2-acrylamido-4-methylpentanamido)phenyl)amino)pyrimi-

-din-5-yl)-2-methyl-5-(3-(trifluoromethyl)benzamido)benzamide (**6**).

***Step1*:**

N-(2-((3-aminophenyl)amino)pyrimidin-5-yl)-2-methyl-5-(3-(trifluoromethyl)

benzamido)benzamide(Intermwdiate1) (0.101g, 0.2mmol) was dissolved in 2ml dry DMF, cooled to 0℃, followed by addition of Et_3_N(0.14ml, 1mmol), HOBt(0.032g, 0.24mmol), N-(tert-Butoxycarbonyl)-L-leucine(0.069g, 0.3mmol), and slowly EDCI(0.077g, 0.4mmol) at 0℃. The reaction was allowed to room temperature slowly and reacted overnight. After stopping the reaction, the volatile components were removed under reduced pressure. The residue was diluted with saturated aqueous sodium bicarbonate and ethyl acetate. The organic phase was washed with brine, dried over anhydrous sodium sulfate, concentrated and purified by column chromatography (gradient: 30-50% EtOAc in hexanes) to yield (S)-tert-butyl (4-methyl-1-((3-((5-(2-methyl-5-(3-(trifluoromethyl)benzamido)benzamido)pyrimidin-2-yl)amino)phenyl)amino)-1-oxopentan-2-yl)carbamate (0.105g) as light yellow solids.

***Step2*:**

(S)-tert-butyl(4-methyl-1-((3-((5-(2-methyl-5-(3-(trifluoromethyl)benzamido)

benzamido)pyrimidin-2-yl)amino)phenyl)amino)-1-oxopentan-2-yl)carbamate (0.105g, 0.15mmol) was dissolved in 6ml DCM, treated with 3ml TFA. The mixture was stirred at room temperature for 2 h and concentrated in vacuum. This crude product(~0.17mmol) was dissolved in 2ml THF, cooled to 0℃, added DIEA(56uL,0.34mmol), 1ml water, and followed by a slow addition of acryloyl choloride(21uL, 0.25mmol). The reaction was allowed to room temperature, reacted for 2 hours. After stopping reaction, the volatile components were removed under reduced pressure. The residue was diluted with saturated aqueous sodium bicarbonate and ethyl acetate. The organic phase was washed with brine, dried over anhydrous sodium sulfate, concentrated and purified by column chromatography (gradient: 30-60% EtOAc in hexanes) to yield (S)-N-(2-((3-(2-acrylamido-4-methylpentanamido)

phenyl) amino)pyrimidin-5-yl)-2-methyl-5-(3-(trifluoromethyl)benzamido)benzamide

(**6**) (40.8mg, 30% over three steps) as white solids. ^1^H NMR (400 MHz, DMSO) δ = 10.60 (d, *J*=19.5, 1H), 10.42 (s, 1H), 10.13 (s, 1H), 9.64 (s, 1H), 8.81 (s, 2H), 8.44 – 8.19 (m, 3H), 8.17 – 7.73 (m, 5H), 7.43 – 7.08 (m, 4H), 6.36 (dd, *J*=17.1, 10.2, 1H), 6.10 (dd, *J*=17.1, 2.1, 1H), 5.60 (dd, *J*=10.2, 2.1, 1H), 4.61 (dd, *J*=14.2, 8.6, 1H), 2.44 – 2.28 (m, 3H), 1.56 (ddt, *J*=35.0, 22.0, 6.9, 3H), 0.91 (dd, *J*=11.7, 6.4, 6H). ^13^C NMR (100 MHz, DMSO) δ = 171.51, 167.99, 164.89, 164.41, 156.99, 150.25, 141.35, 139.62, 136.98, 135.93, 132.31, 131.95, 131.46, 131.34, 130.29, 129.82, 129.51, 129.01, 128.73, 126.80, 125.99, 125.75, 124.63, 123.07, 123.03, 122.25, 119.72, 114.34, 113.02, 109.97, 52.32, 41.51, 24.86, 23.45, 22.12, 19.27. HRMS-ESI calcd. for C35H34F3N7NaO4 [M+Na^+^]: 696.2522; Found: 696.2516.

(S)-N-(2-((3-(3-acrylamidopropanamido)phenyl)amino)pyrimidin-5-yl)-2-methyl-5-(3-(trifluoromethyl)benzamido)benzamide(**3**). white solids. ^1^H NMR (400 MHz, DMSO) δ = 10.58 (s, 1H), 10.42 (s, 1H), 9.90 (s, 1H), 9.63 (s, 1H), 8.80 (s, 2H), 8.35 – 8.17 (m, 3H), 8.06 – 7.74 (m, 5H), 7.32 (t, *J*=9.1, 3H), 7.16 (t, *J*=8.1, 1H), 6.23 (dd, *J*=17.1, 10.1, 1H), 6.07 (dd, *J*=17.1, 2.3, 1H), 5.57 – 5.51 (m, 1H), 3.41 (dd, *J*=12.6, 6.5, 2H), 2.52 (dd, *J*=12.3, 5.5, 2H),, 2.38 (s, 3H). ^13^C NMR (101 MHz, DMSO) δ = 169.74, 167.99, 165.12, 164.40, 157.00, 150.25, 141.33, 139.82, 136.97, 135.93, 132.30, 132.22, 131.46, 131.33, 130.29, 129.82, 129.50, 128.96, 128.73, 126.77, 125.78, 125.44, 124.59, 123.07, 122.23, 119.70, 114.15, 112.91, 109.80, 36.66, 35.53, 19.28. HRMS-ESI calcd. for C32H28F3N7NaO4 [M+Na^+^]: 654.2053; Found: 654.2058.

(S)-1-acryloyl-N-(3-((5-(2-methyl-5-(3-(trifluoromethyl)benzamido)benzamido)

pyrimidin-2-yl)amino)phenyl)pyrrolidine-2-carboxamide(**4**). white solids. ^1^H NMR (400 MHz, DMSO) δ = 10.59 (s, 1H), 10.43 (s, 1H), 10.08 (d, *J*=51.0, 1H), 9.65 (d, *J*=10.3, 1H), 8.81 (s, 2H), 8.37 – 8.23 (m, 2H), 8.05 – 7.95 (m, 2H), 7.93 (s, 1H), 7.89 – 7.73 (m, 2H), 7.41 – 7.25 (m, 3H), 7.18 (q, *J*=7.7, 1H), 6.49 (ddd, *J*=125.7, 16.8, 10.2, 1H), 6.13 (d, *J*=16.6, 1H), 5.67 (dd, *J*=21.3, 11.0, 1H), 4.61 (dd, *J*=57.6, 5.2, 1H), 3.65 (dd, *J*=29.9, 21.2, 2H), 2.38 (s, 3H), 2.04 (dd, *J*=37.3, 30.3, 2H), 1.91 (s, 2H). ^13^C NMR (101 MHz, DMSO) δ = 163.20, 163.13, 160.23, 156.65, 156.30, 156.02, 149.23, 142.50, 133.65, 133.57, 132.03, 129.21, 128.17, 124.55, 123.69, 123.59, 122.53, 122.14, 122.06, 121.74, 121.67, 121.60, 121.22, 120.97, 119.90, 119.02, 118.02, 116.87, 116.84, 115.31, 115.26, 114.48, 111.96, 106.73, 106.42, 105.28, 105.10, 102.16, 102.01, 52.88, 52.62, 39.70, 39.44, 24.65, 22.18, 17.11, 14.96, 11.52. HRMS-ESI calcd. for C34H30F3N7NaO4 [M+Na^+^]: 680.2209; Found: 680.2204.

(S)-N-(2-((3-(2-acrylamidopropanamido)phenyl)amino)pyrimidin-5-yl)-2-methyl-5-(3-(trifluoromethyl)benzamido)benzamide(**5**). white solids. ^1^H NMR (400 MHz, DMSO) δ = 10.57 (s, 1H), 10.42 (s, 1H), 10.04 (s, 1H), 9.65 (s, 1H), 8.81 (s, 2H), 8.42 (d, *J*=7.4, 1H), 8.35 – 8.23 (m, 2H), 8.10 – 7.74 (m, 5H), 7.26 (dq, *J*=49.2, 8.1, 4H), 6.36 (dd, *J*=17.1, 10.2, 1H), 6.10 (dd, *J*=17.1, 2.1, 1H), 5.60 (dd, *J*=10.2, 2.1, 1H), 4.56 (p, *J*=7.0, 1H), , 2.35 (d, *J*=22.6, 3H), 1.32 (d, *J*=7.0, 3H). ^13^C NMR (101 MHz, DMSO) δ = 171.56, 167.99, 164.69, 164.41, 156.98, 150.23, 141.38, 139.63, 136.97, 135.93, 132.30, 131.95, 131.45, 131.33, 130.29, 129.81, 129.50, 129.02, 126.81, 125.95, 124.60, 123.07, 122.24, 119.71, 114.30, 112.90, 109.87, 49.42, 19.27, 18.78. HRMS-ESI calcd. for C32H28F3N7NaO4 [M+Na^+^]: 654.2053; Found: 654.2058.

(S)-N-(2-((3-(2-acrylamido-3-phenylpropanamido)phenyl)amino)pyrimidin-5-yl)-2-methyl-5-(3-(trifluoromethyl)benzamido)benzamide(**7**). white solids. ^1^H NMR (400 MHz, DMSO) δ = 10.58 (s, 1H), 10.43 (s, 1H), 10.19 (s, 1H), 9.67 (s, 1H), 8.81 (s, 2H), 8.54 (d, *J*=8.4, 1H), 8.37 – 8.20 (m, 2H), 7.98 (dd, *J*=24.2, 16.3, 3H), 7.88 – 7.74 (m, 2H), 7.43 – 7.09 (m, 9H), 6.31 (dd, *J*=17.0, 10.3, 1H), 6.03 (d, *J*=17.1, 1H), 5.56 (d, *J*=10.3, 1H), 4.81 (d, *J*=4.7, 1H), 3.07 (dd, *J*=13.6, 4.4, 1H), 2.88 (dd, *J*=13.3, 9.9, 1H), 2.32 (d, *J*=48.8, 3H). ^13^C NMR (101 MHz, DMSO) δ = 170.53, 168.00, 164.91, 164.41, 156.97, 150.23, 141.39, 139.45, 138.14, 136.98, 135.93, 132.31, 131.85, 131.46, 131.34, 130.29, 129.68, 129.64, 129.47, 129.04, 128.53, 126.83, 126.08, 124.64, 119.72, 114.41, 113.04, 109.97, 55.30, 38.30, 19.28. HRMS-ESI calcd. for C38H32F3N7NaO4 [M+Na^+^]: 730.2366; Found: 730.2366.

**Preparation of compound 8-12**

Synthesis of Trifluoroacetate of (S)-N-(2-((3-(2-acrylamido-6-aminohexanamido)phenyl)amino)

pyramidin-5-yl)-2-methyl-5-(3-(trifluoromethyl)benzamido)benzamide(**11**).

***Step1*:**

N-(2-((3-aminophenyl)amino)pyrimidin-5-yl)-2-methyl-5-(3-(trifluoromethyl)

benzamido)benzamide(Intermediate 1) (0.07g, 0.14mmol), Nα- [(9H-fluoren-9-ylmethoxy) carbonyl]-Nε- (tert- butoxycarbonyl)-L-lysine(0.101g, 0.21mmol),

HATU(0.105g, 0.28mmol) were dissolved in dry DMF, cooled to 0℃, then added DIEA(46uL, 0.28mmol). The reaction was allowed to room temperature slowly and reacted overnight. While stopping the reaction, the volatile components were removed under reduced pressure. The residue was diluted with saturated aqueous sodium bicarbonate and ethyl acetate. The organic phase was washed with brine, dried over anhydrous sodium sulfate, concentrated and purified by column chromatography (gradient: 30-60% EtOAc in hexanes) to yield (S)-(9H-fluoren-9-yl)methyl tert-butyl (6-((3-((5-(2-methyl-5-(3-(trifluoromethyl)benzamido)benzamido)pyrimidin-2-yl)

amino)phenyl)amino)-6-oxohexane-1,5-diyl)dicarbamate (0.128g, 96%) as yellow solids.

***Step2*:**

(S)-(9H-fluoren-9-yl)methyl tert-butyl (6-((3-((5-(2-methyl-5-(3-(trifluoromethyl)

benzamido)benzamido)pyrimidin-2-yl)amino)phenyl)amino)-6-oxohexane-1,5-diyl)dicarbamate (0.128g, 0.13mmol) was dissolved in 1ml dry DMF, treated with 1ml morpholine, reacted at room temperature for 3 hours. While stopping the reaction, the volatile components were removed under reduced pressure. The residue was diluted with saturated aqueous sodium bicarbonate and ethyl acetate. The organic phase was washed with brine, dried over anhydrous sodium sulfate, concentrated and purified by column chromatography (gradient: 2-4% MeOH in DCM) to yield (S)-tert-butyl (5-amino-6-((3-((5-(2-methyl-5-(3-(trifluoromethyl)benzamido)

benzamido)pyrimidin-2-yl)amino)phenyl)amino)-6-oxohexyl)carbamate(0.084g, 88%) as light yellow solids.

***Step3:***

(S)-tert-butyl (5-amino-6-((3-((5-(2-methyl-5-(3-(trifluoromethyl)benzamido)benza-

-mido)pyrimidin-2-yl)amino)phenyl)amino)-6-oxohexyl)carbamate(0.084g, 0.114mmol) was dissolved in 1ml THF, cooled to 0℃, added DIEA(38uL,0.23mmol), 0.5ml water, and followed by a slow addition of acryloyl choloride(18uL, 0.23mmol). The reaction was allowed to room temperature, reacted for 2 hours. While stopping reaction, the volatile components were removed under reduced pressure. The residue was diluted with saturated aqueous sodium bicarbonate and ethyl acetate. The organic phase was washed with brine, dried over anhydrous sodium sulfate, concentrated, yielding crude product(0.080g) as yellow solids. The latter was dissolved in 1ml DCM, treated with 0.5ml TFA. The mixture was stirred at room temperature for 2 h and concentrated in vacuum, the obtained solids was washed with diethyl ether, then gained the title compound(**11**) (0.037 g, 47% over 2 steps) as yellow solids. ^1^H NMR (400 MHz, DMSO) δ = 10.60 (s, 1H), 10.44 (s, 1H), 10.11 (s, 1H), 9.64 (s, 1H), 8.80 (s, 2H), 8.39 (d, *J*=7.9, 1H), 8.31 (s, 1H), 8.27 (d, *J*=7.9, 2H), 8.04 (s, 1H), 7.97 (d, *J*=7.8, 2H), 7.93 (d, *J*=2.1, 1H), 7.88 – 7.75 (m, 3H), 7.37 – 7.26 (m, 4H), 7.17 (t, *J*=8.1, 1H), 6.37 (dd, *J*=17.1, 10.2, 1H), 6.10 (dd, *J*=17.1, 2.1, 1H), 5.60 (dd, *J*=10.2, 2.1, 1H), 4.51 (dd, *J*=13.8, 8.3, 1H), 2.35 (d, *J*=22.7, 3H), 1.76 – 1.53 (m, 2H), 1.35 (dd, *J*=16.0, 10.2, 4H). ^13^C NMR (75 MHz, DMSO) δ = 171.19, 168.02, 164.99, 164.45, 156.97, 150.26, 141.37, 139.60, 136.96, 135.92, 132.32, 131.94, 131.49, 131.38, 130.32, 129.88, 129.45, 129.06, 128.75, 126.80, 126.18, 126.04, 124.65, 122.62, 122.27, 119.74, 114.32, 112.94, 109.89, 100.00, 53.92, 41.71, 32.97, 32.58, 23.36, 19.29. HRMS-ESI calcd. for C35H36F3N8O4 [M+H^+^]: 689.2812; Found: 689.2807.

(S)-N-(2-((3-(2-acrylamido-3-hydroxypropanamido)phenyl)amino)pyrimidin-5-yl)-2-methyl-5-(3-(trifluoromethyl)benzamido)benzamide(**8**). Light yellow solids. ^1^H NMR (400 MHz, DMSO) δ = 10.58 (s, 1H), 10.43 (s, 1H), 10.05 (s, 1H), 9.65 (s, 1H), 8.81 (s, 2H), 8.30 (dd, *J*=12.8, 7.6, 3H), 8.09 (s, 1H), 7.98 (d, *J*=7.5, 1H), 7.93 (s, 1H), 7.89 – 7.69 (m, 2H), 7.33 (dd, *J*=15.7, 7.4, 3H), 7.18 (t, *J*=8.1, 1H), 6.43 (dd, *J*=17.1, 10.2, 1H), 6.11 (d, *J*=17.3, 1H), 5.61 (d, *J*=11.3, 1H), 5.06 (t, *J*=5.2, 1H), 4.60 (dd, *J*=13.1, 5.9, 1H), 3.66 (t, *J*=5.3, 2H), 2.38 (s, 3H). ^13^C NMR (101 MHz, DMSO) δ = 169.32, 168.00, 164.98, 164.42, 156.99, 150.24, 141.34, 139.60, 136.97, 135.93, 132.31, 132.06, 131.46, 131.34, 130.29, 129.83, 129.51, 128.96, 128.73, 126.79, 125.97, 125.78, 124.63, 124.60, 123.07, 122.24, 119.72, 114.35, 112.99, 109.96, 62.26, 56.25, 19.27. HRMS-ESI calcd. for C32H28F3N7NaO5 [M+Na^+^]: 670.2002; Found: 670.1999.

(S)-2-acrylamido-N1-(3-((5-(2-methyl-5-(3- (trifluoromethyl)benzamido)benzamido) pyrimidin-2-yl)amino)phenyl)succinamide(**9**). Light yellow solids. ^1^H NMR (400 MHz, DMSO) δ = 10.68 (s, 1H), 10.47 (s, 1H), 10.08 (s, 1H), 9.62 (s, 1H), 8.81 (s, 2H), 8.49 (s, 1H), 8.32 (d, *J*=11.1, 2H), 8.06 (s, 1H), 7.97 (d, *J*=12.3, 2H), 7.86 (d, *J*=8.4, 1H), 7.79 (t, *J*=7.8, 1H), 7.42 (s, 1H), 7.33 (t, *J*=9.6, 2H), 7.27 (d, *J*=7.8, 1H), 7.16 (t, *J*=8.0, 1H), 6.92 (s, 1H), 6.34 (dd, *J*=17.1, 10.1, 1H), 6.10 (d, *J*=17.1, 1H), 5.61 (d, *J*=10.1, 1H), 4.80 (d, *J*=6.1, 1H), 2.60 (dd, *J*=15.1, 6.1, 2H), 2.38 (s, 3H). ^13^C NMR (101 MHz, DMSO) δ = 171.57, 170.14, 168.04, 164.92, 164.47, 157.01, 150.30, 141.32, 139.63, 136.95, 135.93, 132.31, 131.91, 131.48, 131.39, 130.32, 129.84, 129.52, 128.98, 128.75, 126.77, 126.16, 125.70, 124.64, 123.08, 122.28, 119.74, 114.39, 113.07, 110.09, 67.48, 51.19, 37.69, 25.58, 19.28. HRMS-ESI calcd. for C33H30F3N8O5 [M+H^+^]: 675.2291; Found: 675.2286.

(S)-4-acrylamido-5-((3-((5-(2-methyl-5-(3- (trifluoromethyl)benzamido)benzamido) pyrimidin-2-yl)amino)phenyl)amino)-5-oxopentanoic acid(**10**). Light yellow solids. ^1^H NMR (400 MHz, DMSO) δ = 12.14 (s, 1H), 10.58 (s, 1H), 10.42 (s, 1H), 10.10 (s, 1H), 9.65 (s, 1H), 8.80 (s, 2H), 8.42 (d, *J*=7.6, 1H), 8.32 (s, 1H), 8.28 (d, *J*=7.8, 1H), 8.04 (s, 1H), 7.98 (d, *J*=7.4, 1H), 7.92 (s, 1H), 7.84 (d, *J*=8.3, 1H), 7.79 (t, *J*=7.7, 1H), 7.35 (dd, *J*=15.5, 8.1, 2H), 7.28 (d, *J*=7.6, 1H), 7.18 (t, *J*=8.2, 1H), 6.37 (dd, *J*=16.9, 10.2, 1H), 6.11 (d, *J*=17.1, 1H), 5.62 (d, *J*=10.3, 1H), 4.60 – 4.48 (m, 1H), 2.38 (s, 3H), 2.34 – 2.19 (m, 2H), 2.04 – 1.79 (m, 2H). ^13^C NMR (101 MHz, DMSO) δ = 174.42, 170.53, 168.03, 165.09, 164.46, 156.98, 150.29, 141.39, 139.46, 136.96, 135.93, 132.31, 131.83, 131.48, 131.39, 130.32, 129.84, 129.52, 129.08, 128.78, 126.81, 126.22, 125.78, 124.64, 123.08, 122.28, 119.74, 114.44, 113.04, 109.99, 53.33, 31.04, 28.12, 19.29. HRMS-ESI calcd. for C34H31F3N7O6 [M+H^+^]: 690.2288; Found: 690.2283.

(S)-N-(2-((3-(2-acrylamido-3-(1H-imidazol-5-yl)propanamido)phenyl)amino)

pyrimidin-5-yl)-2-methyl-5-(3-(trifluoromethyl)benzamido)benzamide(**12**). Light yellow solids. ^1^H NMR (400 MHz, DMSO) δ = 10.70 (s, 1H), 10.48 (s, 1H), 10.23 (s, 1H), 9.63 (s, 1H), 8.81 (s, 2H), 8.59 (d, *J*=7.6, 1H), 8.32 (d, *J*=8.7, 2H), 8.06 (s, 1H), 7.95 (t, *J*=11.9, 3H), 7.87 (d, *J*=8.2, 1H), 7.79 (t, *J*=7.5, 1H), 7.42 – 7.22 (m, 3H), 7.17 (t, *J*=8.1, 1H), 6.96 (s, 1H), 6.36 (dd, *J*=17.4, 10.1, 1H), 6.08 (d, *J*=16.9, 1H), 5.60 (d, *J*=10.2, 1H), 4.79 (d, *J*=6.1, 1H), 3.11 – 2.83 (m, 2H), 2.38 (s, 3H). ^13^C NMR (101 MHz, DMSO) δ = 170.08, 167.97, 164.91, 164.37, 156.90, 150.23, 141.27, 139.42, 136.92, 135.83, 134.67, 132.29, 131.82, 131.33, 131.26, 130.20, 129.66, 129.40, 128.90, 128.67, 126.74, 126.03, 125.68, 124.65, 122.24, 119.74, 114.38, 113.02, 110.05, 53.87, 29.41, 19.23. HRMS-ESI calcd. for C35H31F3N9O4 [M+H^+^]: 698.2451; Found: 698.2447.

**Preparation of compound 13-14**

**Synthesis of** **fluorescence probe**, (S)-3-(3-((5-acrylamido-6-((3-((5-(2-methyl-5-(3-

(trifluoromethyl)benzamido)benzamido)pyrimidin-2-yl)amino)phenyl)amino)-6-oxohexyl)amino)-3-oxopropyl)-5,5-difluoro-7,9-dimethyl-5H-dipyrrolo[1,2-c:2',1'-f][1,3,2]diazaborinin-4-ium-5-uide(**14**).

**11** (0.038g, 0.055mmol), 4,4-difluoro-5,7-dimethyl-4-bora-3a,4a -diaza-(S)-indacene-3- propionic acid (BODIPY(R) FL) (0.015g, 0.05mmol), HATU(0.038g, 0.1mmol) were dissolved in dry DMF, cooled to 0℃, then added DIEA(41uL, 0.25mmol). The reaction was allowed to room temperature slowly and reacted overnight. While stopping the reaction, the volatile components were removed under reduced pressure. The residue was diluted with saturated aqueous sodium bicarbonate and ethyl acetate. The organic phase was washed with brine, dried over anhydrous sodium sulfate, concentrated and purified by column chromatography (gradient: 60% EtOAc in hexanes-1% MeOH in EtOAc) to yield the designed product(**14**) (0.033g, 70%) as red solids. ^1^H NMR (400 MHz, MeOD) δ = 8.77 (s, 2H), 8.28 (s, 1H), 8.22 (d, *J*=7.9, 1H), 8.08 (s, 1H), 7.95 (d, *J*=2.0, 1H), 7.90 (d, *J*=7.7, 1H), 7.75 (d, *J*=7.8, 1H), 7.73 – 7.66 (m, 1H), 7.38 (s, 1H), 7.34 (t, *J*=6.6, 2H), 7.24 (dt, *J*=15.7, 8.0, 2H), 6.97 (d, *J*=3.9, 1H), 6.38 (dd, *J*=17.1, 10.1, 1H), 6.25 (dd, *J*=17.1, 1.7, 2H), 6.18 (s, 1H), 5.69 (dd, *J*=10.2, 1.6, 1H), 4.58 – 4.49 (m, 1H), 3.35 (s, 2H), 3.20 (dd, *J*=13.5, 6.6, 4H), 2.57 (t, *J*=7.6, 2H), 2.49 (s, 3H), 2.47 (s, 3H), 2.26 (s, 3H), 1.95 – 1.85 (m, 1H), 1.80 (m, 1H), 1.55 (dd, *J*=13.6, 6.6, 2H), 1.50-1.37 (m, 2H), 1.36 – 1.27 (m, 2H). ^13^C NMR (101 MHz, MeOD) δ = 165.68, 163.88, 163.43, 162.32, 161.59, 158.86, 157.86, 155.22, 152.07, 149.34, 142.68, 136.58, 132.94, 130.70, 128.40, 128.34, 127.89, 127.18, 125.67, 124.41, 123.31, 123.18, 123.01, 122.48, 121.53, 120.76, 120.46, 120.29, 118.15, 117.46, 116.56, 116.36, 114.87, 112.13, 111.91, 108.47, 107.12, 105.99, 102.99, 46.35, 30.86, 26.82, 23.80, 20.86, 16.47, 15.01, 10.09, 5.67, 2.00. HRMS-ESI calcd. for C49H48BF5N10NaO5 [M+Na^+^]: 985.3720; Found: 985.3717.

(S)-N-(2-((3-(2-acrylamido-6-(pent-4-ynamido)hexanamido)phenyl)amino)pyrimidin-5-yl)-2-methyl-5-(3-(trifluoromethyl)benzamido)benzamide (**13**). Light yellow solids. ^1^H NMR (400 MHz, DMSO) δ = 10.58 (s, 1H), 10.42 (s, 1H), 10.09 (s, 1H), 9.64 (s, 1H), 8.81 (s, 2H), 8.41 – 8.23 (m, 3H), 8.08 – 7.90 (m, 3H), 7.90 – 7.74 (m, 3H), 7.44 – 7.25 (m, 3H), 7.18 (t, *J*=8.1, 1H), 6.39 (dd, *J*=17.1, 10.2, 1H), 6.11 (dd, *J*=17.1, 2.0, 1H), 5.61 (dd, *J*=10.2, 2.0, 1H), 4.52 (dd, *J*=13.5, 8.4, 1H), 3.09 – 2.96 (m, 2H), 2.72 (t, *J*=2.5, 1H), 2.39 (s, 3H), 2.37 – 2.28 (m, 2H), 2.23 (t, *J*=7.0, 2H), 1.79 – 1.57 (m, 2H), 1.50 – 1.16 (m, 4H). ^13^C NMR (101 MHz, DMSO) δ = 171.11, 170.52, 168.02, 165.00, 164.43, 157.00, 150.27, 141.39, 139.59, 136.98, 135.95, 132.31, 131.97, 131.47, 131.36, 130.30, 129.85, 129.53, 129.04, 128.77, 126.81, 126.00, 125.79, 124.61, 123.06, 122.26, 119.74, 114.35, 112.98, 109.94, 84.23, 71.69, 53.80, 38.81, 34.69, 32.41, 29.31, 23.40, 19.28, 14.75. HRMS-ESI calcd. for C40H39F3N8NaO5 [M+Na^+^]: 791.2893; Found: 791.2889.

1. **NMR and HPLC Spectra of New Compounds**

**4.1 NMR Spectra**

Compound **3**

Compound **4**

Compound **5**

Compound **6**

Compound **7**

Compound **8**

Compound **9**

Compound **10**

Compound **11**

Compound **12**

Compound **13**

Fluorescence probe **14**

**4.2 HPLC spectra**

Compound **3**


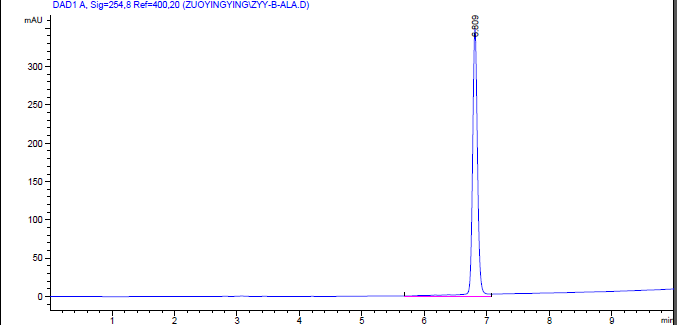


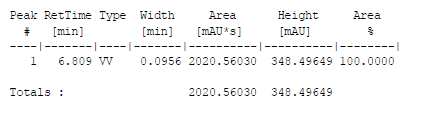


Compound **4**


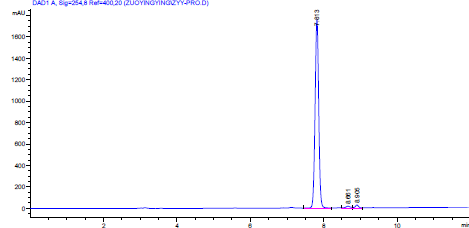


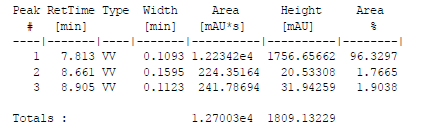


Compound **5**


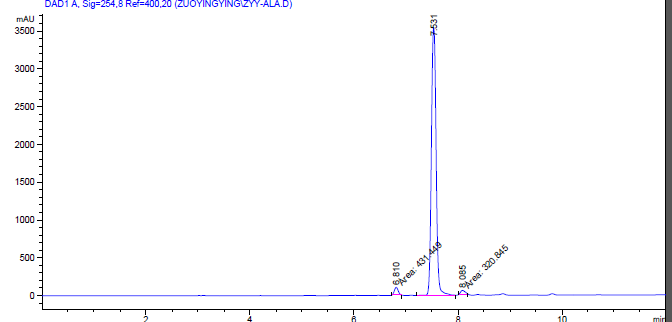


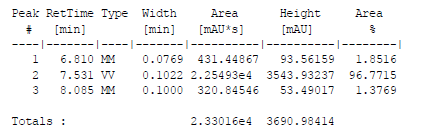


Compound **6**


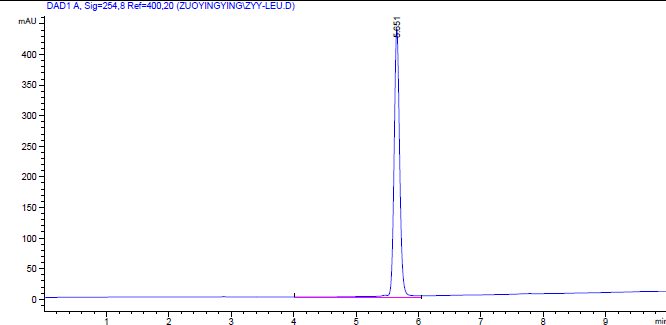


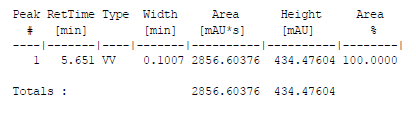


Compound **7**


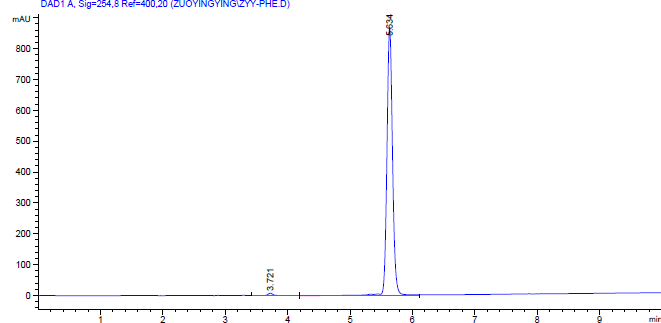


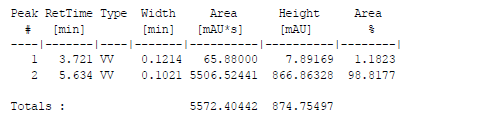


Compound **8**


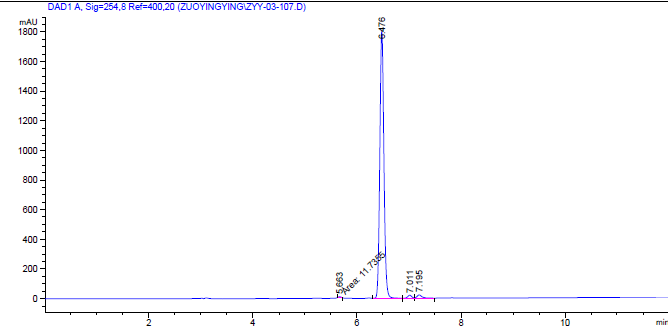


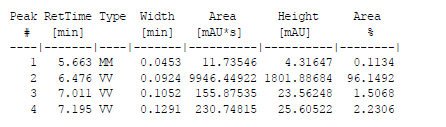


Compound **9**


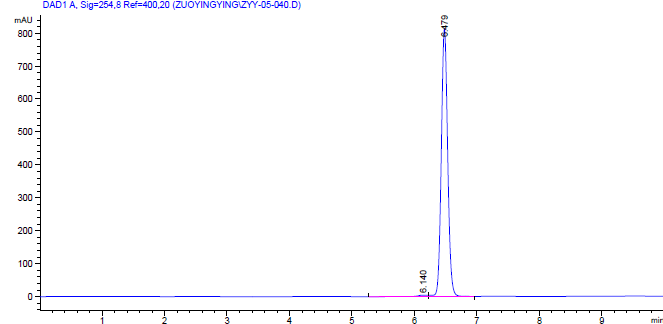


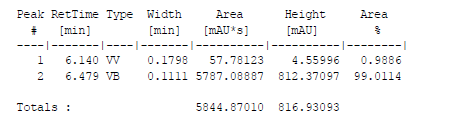


Compound **10**


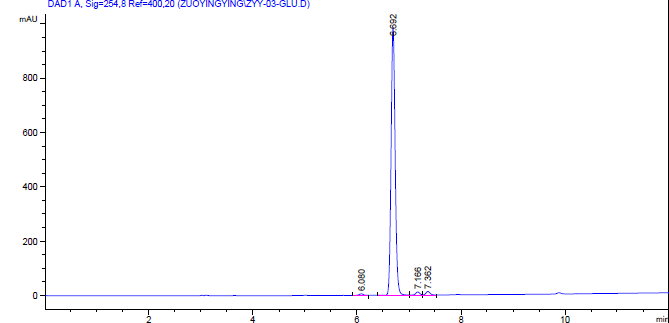


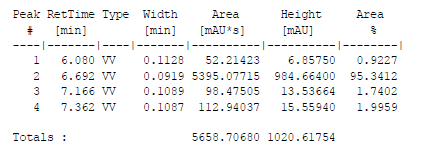


Compound **11**


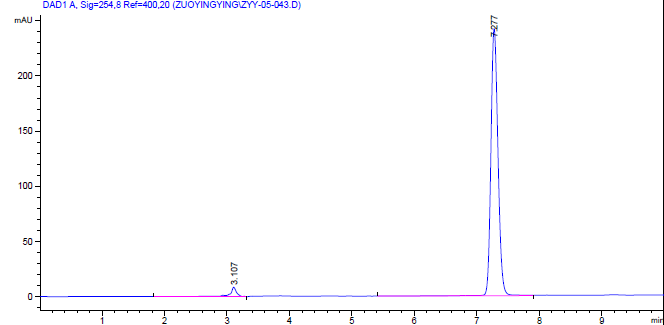


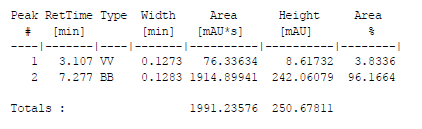


Compound **12**


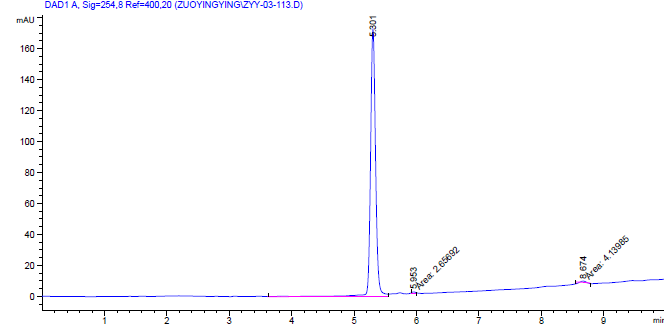


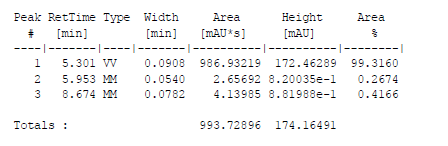


Compound **13**


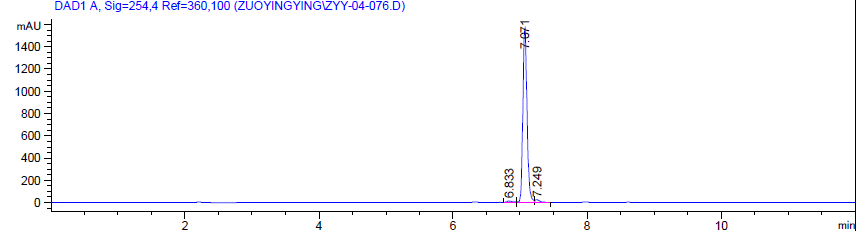


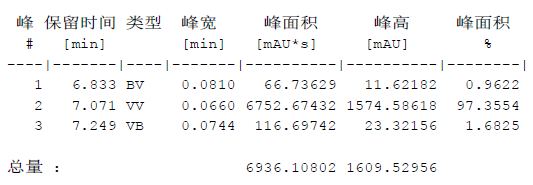


Compound **14**


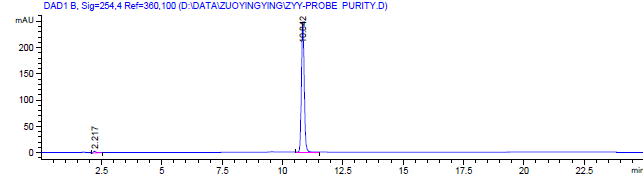


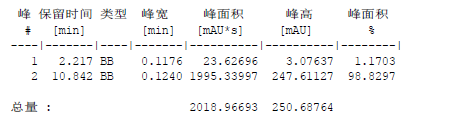


1. **Biological data**

**Supplementary figure S1**: competition experiment in OCI-Ly7 cells (a) and Jurkat cells (b). 1.5×10^6^ cells were pre-incubated with compounds for 1h at 1μM before labeling with probe **14** for 2h at 0.5μM. Then, lysed, quantified and analyzed by SDS/PAGE and fluorescent gel scanning (fluorescence, CY2).

**Supplementary figure S2**: Btk labeling in OCI-Ly7 cells by PCI-33380. (a) Concentration-dependent labeling of Btk in OCI-Ly7 cells. (b) time-dependent labeling of Btk in OCI-Ly7 cells. (c) measurement of the extent of Btk occupancy by inhibitors (ibrutinib and compound **2**) in live cells.
